# PhiC31-based Site-Specific Transgenesis System for Production of Transgenic Bovine Embryos by Somatic Cell Nuclear Transfer and Intracytoplasmic Sperm Injection

**DOI:** 10.22074/cellj.2018.4385.

**Published:** 2018-01-01

**Authors:** Mohammad Hadi Sekhavati, Sayed Morteza Hosseini, Mojtaba Tahmoorespur, Kamran Ghaedi, Farnoosh Jafarpour, Mehdi Hajian, Kyanoosh Dormiani, Mohammad Hossain Nasr-Esfahani

**Affiliations:** 1 Department of Animal Science, Ferdowsi University of Mashhad, Mashhad, Iran; 2 Department of Reproductive Biotechnology, Reproductive Biomedicine Research Center, Royan Institute for Biotechnology, ACECR, Isfahan, Iran; 3 Department of Biology, Facualty of Sciences, Uneversity of Isfahan, Isfahan, Iran; 4 Department of Cellular Biotechnology, Cell Science Research Center, Royan Institute for Biotechnology, ACECR, Isfahan, Iran

**Keywords:** Intracytoplasmic Sperm Injection, Somatic Cell Nuclear Transfer, Transgenesis

## Abstract

**Objective:**

The Streptomyces phage phiC31 integrase offers a sequence-specific method of transgenesis with a robust long-term gene expression. PhiC31 has been successfully developed in a variety of tissues and organs for purpose of *in vivo* gene therapy. The objective of the present experiment was to evaluate PhiC31-based site-specific transgenesis system for production of transgenic bovine embryos by somatic cell nuclear transfer and intracytoplasmic sperm injection.

**Materials and Methods:**

In this experimental study, the application of phiC31 integrase system was evaluated for generating transgenic bovine embryos by somatic cell nuclear transfer (SCNT) and sperm mediated gene transfer (SMGT) approaches.

**Results:**

PhiC31 integrase mRNA and protein was produced *in vitro* and their functionality was confirmed. Seven phiC31 recognizable bovine pseudo attachment sites of phage (attP) sites were considered for evaluation of site specific recombination. The accuracy of these sites was validated in phic31 targeted bovine fibroblasts using polymerase chain reaction (PCR) and sequencing. The efficiency and site-specificity of phiC31 integrase system was also confirmed in generated transgenic bovine embryo which successfully obtained using SCNT and SMGT technique.

**Conclusion:**

The results showed that both SMGT and SCNT-derived embryos were enhanced green fluorescent protein (*EGFP*) positive and phiC31 integrase could recombine the reporter gene in a site specific manner. These results demonstrate that attP site can be used as a proper location to conduct site directed transgenesis in both mammalian cells and embryos in phiC31 integrase system when even combinaed to SCNT and intracytoplasmic sperm injection (ICSI) method.

## Introduction

Genetically engineered (transgenic) animals hold promising applications in biomedicine and agriculture. Recently, transgenic animal models have become a key tool in functional genomics to understand the initiation and perpetuation of human diseases. Moreover, they are an invaluable system for large scale production of therapeutic proteins (1, 2). The contemporary methods that are used for production of transgenic animals include intra-pronuclear zygotic DNA microinjection and somatic cell nuclear transfer (SCNT). DNA microinjection into the male pronucleus of a zygote is well-established in rodents (3), SCNT is an obvious choice of transgene delivery method in farms mammalian species because their zygotes are optically opaque, due to the presence of lipid granules in the cytoplasm; which makes the pronuclear microinjection difficult and inefficient (4, 5). Recent developments in studies of sperm-mediated gene transfer (SMGT) suggested that sperm cells can be considered vectors to transfer DNA into the oocyte during *in vitro* fertilization (IVF) or intra cytoplasmic sperm injection (ICSI) (6-8), but also suggests that the final fate of the exogenous sequences transferred by sperm is not always predictable (6). Since the highly condensed structure of sperm chromatin makes it virtually inaccessible to foreign molecules, we previously showed that *in vitro* decondensation of bovine sperm with heparin and glutathione (GSH) not only remarkably increase the efficiency of ICSI, but also provided new insights for in vitro transfection of sperm cells before being used for SMGT (9). 

Classical methods for generating of transgenic animals usually integrates an uncontrolled number of transgene copies into random genomic sites (10). Transgenes which generated by this method are additionally susceptible to transgene silencing due to position site-dependent effects or tumor activation which caused by transgenesis near to oncogenes (11, 12). Although retroviruses and transposons would improve the efficiency of single-copy transgenesis, uncontrollability of the integration copy number is still a major limitation (13). Homologous recombination targets the transgene to a specific genomic site, but targeting loci by homologous recombination in technically demanding and time consuming (14) and the final efficiency is presently extremely low in mammalian cells (15). These problems can be overcome by recently developed hybrid nuclease technologies including zinc-finger (ZFN), transcription activator-like effector nuclease (TALEN) and CRISPR associated protein 9 (Cas9), but it is still challenging to screen nucleases with high affinity and specificity (16). Therefore, developing of an alternative molecular tools which introduce site-specific transgene integration with robust gene expression are still a problem for site-directed transgenesis. 

The streptomyces phage PhiC31 integrase has been used as a powerful tool to carry out irreversible and unidirectional recombination between attachment sites of phage (attP) and bacteria (attB) genomes (17). Interestingly, these prokaryotederived integrases have been successfully used to target transgene to specific sites in eukaryotic cells of several species (2, 14, 18-21). This system can integrate the whole plasmid harboring attB sequence into the preferred locations in mammalian genome which so called pseudo attP reviewed by Calos (22). Pseudo sites are naturally present in the region of open chromatin (23). Transgene expression in these sites is robust compared to random integration (24, 25). Importantly, the number of pseudo attP sites is estimated in the range of 100-1000 sites in mammalian genome (26, 27). For example, in bovine genome, 36 pseudo attP sites have been recognized so far by phiC31 integrase system (28-31). As bovine is an economically important farm animal, we introduced three new attP site within bovine genome and then demonstrated that these new pseudo attP sites are in favor of enhanced green fluorescent protein (*EGFP*) transgene expression in bovine embryos produced by SCNT and SMGT. 

## Materials and Methods

In this experimental study, unless otherwise specified, all chemicals and media were obtained from Sigma Chemical Co. (St. Louis, MO, USA) and Gibco (Grand Island, NY, USA), respectively. All animal experiments and procedures described in this study were approved by the Royan Institute Animal Ethics Committee (No. R-084-2003).

## Vector construction

The pCMVInt and the pBCPB^+^ vectors were kindly gifted by Professor M.P. Calos (Stanford University). The pCMVInt contained a phiC31-cDNA site sequence and pBCPB^+^ contained att site sequence. These vectors also contained *EGFP* under the control of the cytomegalovirus (CMV) promoter, the SV40 promoter driving the neomycin (G418)-resistance marker, and the phiC31 attB site. PhiC31 cDNA was cloned into a pET15b vector (Novagen, USA) as follows: pCMVInt vector containing phiC31 was linearized by KpnI and then digested product blunted using klenow fragment (Thermo, USA). In the second step, the cDNA of phiC31 excised from linearized and blunted pCMVint by BamHI. In parallel, PET15b plasmid (Novagen, CA, USA) was linearized and blunted by NdeI and klenow fragment, respectively. Linearized pET15b was digested by BamHI. Finally, linearzied and blunted pET15b backbone and phiC31 open reading frame were gel extracted and ligated using DNA Ligation Kit (Takara, Japan). The pET-phiC31 expression plasmid was amplified in a DH5α strain of *E. coli.* (Invitrogen, USA). We confirmed PhiC31 integrase cDNA by sequencing and also expressed and purified integrase protein in the *E.coli* by using the Ni2^+^-agarose columns (Qiagen, CA) as described previously (20). To construct the pUC19phiC31polyA vector, phiC31 cDNA was amplified by polymerase cjain reaction (PCR) from pCMVInt and then cloned into the pUC19 vector. The PhiC31 integrase cDNA was amplified from the pCMVInt using: 

TP-F:5´-AGCTCTAGAGCTAATACGACTCACTATAG GGAGACCCAAGCTGGCTAGCCACCATGGACACG TACGCGGGTGCTTACG-3´

R:5´-ACGGGATCCCGTTTTTTTTTTTTTTTTTTTTT TTTTTTTTTATTTGTGATCACGCCGCTACGTCTTC CGTGC-3´ primers.

The procedure of PCR is as follows: 94°C for 5 minutes as an initial denaturation step, followed by 27 repetitive cycles at 94°C for 30 seconds, 65°C for 45 seconds, and 72°C for 60 seconds. Final extension of 72°C for 5 minutes was performed at the last stage. PCR products were subjected to electrophoresis on 1% (w/v) agarose (CinnaGen, Iran), purified from agarose gel (Promega, USA) and then cloned into the TA vector (InsTAclone^TM^ PCR Cloning Kit, Thermo, USA). The pTZphiC31polyA construct digested with XbaI enzyme and cloned into an XbaI digested pUC19 (Promega, USA). All restriction enzymes were purchased from Thermo (USA). 

It has been suggested that CMV promoter may be prone to silencing mediated by de novo methylation during zygote genome activation in early embryos (32). To overcome this possible problem, a new vector containing eukaryotic elongation factor 1 alpha driven *EGFP* (EGFPEF1 alpha vector) was constructed by replacing the CMV promoter with EF1 alpha promoter in the pDB2 vector. Briefly, EF1 alpha promoter was amplified using five prime linked primers (Table 1, Table EF1 and EF2 primers) with AseI and NheI restriction enzymes when pBudCE4.1 plasmid (Invitrogen, USA) was used as template in PCR reaction. Subsequently, the full length of EF1 alpha promoter was ligated into the pDB2 plasmid digested by AseI and NheI. 

**Table 1:** The list of primers used in this study


Primer	Primer sequencing (5´-3´)	Reference

EF1	GTTATTAATCGTGAGGCTCCGGT	
EF2	GCCGCTAGCTCACGACACCTGAA	
BF4-F	GCTGGACGTGTAACCCCTTA	(28, 29)
BF4-nest	TGGAATAACGGAGAGACACG	
BF5-F	GGTGCTAGGCATTGCGTTAG	
BF5-nest	TGTGTCTTTGAGGTGCTAGGC	
BF10-F	TTGATACACAGCCTCGCTTG	(28, 29)
BF10-nest	TCCTCACGATTTGCACACTG	
BspF1-F	GCTGGGTGATAGGCACATCT	(28, 29)
BspF1-nest	CAGTGGAGACAACCCAGTGTG	
BspM1-F	CTTCCCAATCCAGAGATCCA	(28, 29)
BpsM1-nest	ATAGAAAGGGGAAATGCGTC	
BUN1-F	TGTGGTTTGTCCAAACTCATC	
BUN1-nest	GCAATTCGGCTTGTCGAC	
BUN2-F	ATCAACTACCGCCACCTCG	
BUN2-nest	GGACCAGATGGGTGAGGTG	
attB-R	GTAGGTCACGGTCTCGAAGC	(28, 29)


## Assessment of the phiC31 protein and mRNA functionality

To assess the phiC31 protein functionality, 1 µg of pB- CBP+ plasmid was incubated with 1 µg purified phiC31 integrase protein in a reaction buffer (pH=8.5, was comprised of 20 mM HEPES 100 mM KCl, 10 mM dithiothreitol, 0.01% bovine serum albumin (BSA) at 30°C for 1 hour. Subsequently, PCR was conducted for screening of site-specific recombination junction using:

F: 5´-GGCGAGAAAGGAAGGGAAGA-3´

R: 5´-ATTAACCCTCACTAAAGGGA-3´

primers. In parallel, for assessment of the phiC31 mRNA activity, PhiC31 RNA and pBCBP+ vector were diluted in microinjection TE buffer to a final concentration of 10:100 ng/µland micro injected into bovine *in vitro* matured (MII) oocytes(n=50) which were prepared as described later in this manuscript. For positive control, pCMVInt was replaced to phiC31mRNA in a parallel micro injection experiment. Injected oocytes were chemically activated and cultured for 48 hours in vitro as described later in this manuscript. Two cell embryos were lysed using CelLytic M (Sigma, USA) and screened forsite-specific recombination using PCR. The process of spermchromatin *in vitro* decondensation was as described previously (9). 

## DNA labeling and incubation of sperm cells with plasmid DNA and *in vitro* decondensation of sperm chromatin

**Fig.1:**
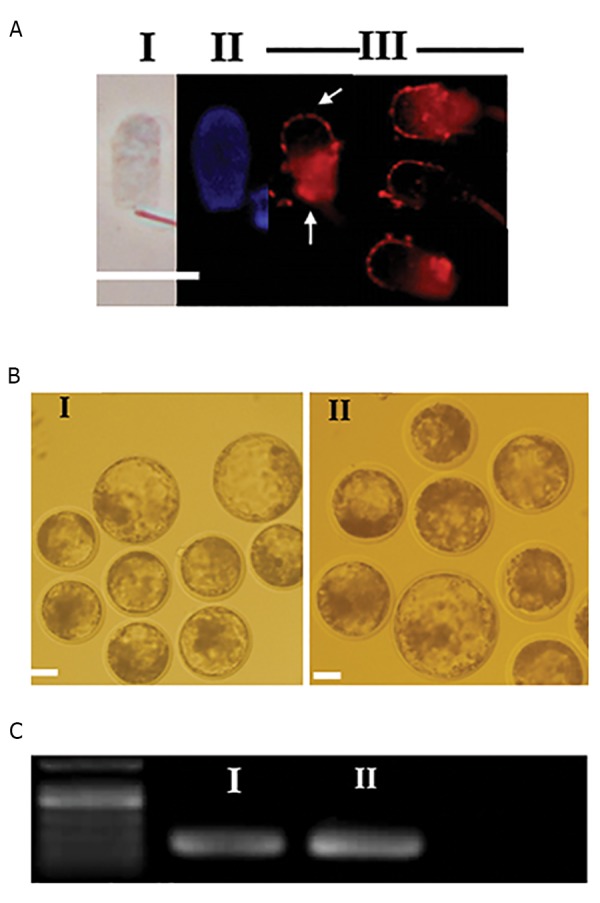
Head sperm DNA uptake, sperm mediated gene transfer and screening for *EGFP* expression in SMGT derived embryos. A. Sperm decondensation and exogenous DNA uptake, I. Phase contrast microscopy observation for decondensedsperm, II. Hoechst staining for decondensedsperm, III. The pattern of DNA uptake by decondensedsperm. This pattern depicted by arrowseither over acrosomal ridge or in the post-acrosomal region, B. SMGT derived embryos, I. Blastocysts formation by injection of sperm which incubatedwith 200 ng pDB2 for 30 minutes at RT, II. Blastocysts formation by injectionof sperm which incubated with 1000 ng pDB2 for 30 minutes at RT, and C. RT-PCR for detection of *EGFP* expressionin SMGT derived embryos. Lane 1: DNA marker, I. SMGT derived embryos obtained by injection of sperm whichincubated by 200 ng pDB2 for 30 minutes at RT, and II. SMGT derived embryosobtained by injection of sperm which incubated by 1000 ng pDB2 for 30 minutes at RT (scale bars: 15 µm). SMGT; Sperm mediated gene transfer and RT-PCR; Real time-polymerase chain reaction.

In order to track the uptake and localization of the DNA bysperm cells, pDB2 plasmid was labeled by CX-Rhodamineusing IT^®^ Tracker™ Intracellular Nucleic Acid Localization Kit (Mirus, USA) according to manufacturer’s guideline (Fig .1). In brief, commercial frozen sperm from three different bulls was thawed and pooled together. Completely motile bovine sperm were obtained by centrifugation of thawed and washed semen over discontinuous layers of PureSperm® gradients. Motile sperm were washed with tissue culture medium 199 (TCM-199) (10 minutes, 1000×g), the sperm pellet resuspended in 30 µl TCM-199 and then a population of 1×10^6^ sperm cells were incubated with 200 ng labeled plasmid for 30 minutes at room temperature (RT). Labeled sperm cells were co-incubated in a new tube containing heparin (80 mM) and GSH (15 mM) for 7 hours at 39°C, 20% O_2_ and 6% CO_2_. DNA-uptake by sperm cells was assessed at ×100 magnification of a fluorescent microscope (Olympus BX51, Japan). Upon exposure to UV light (excitation and emission are needed), a digital image of each sample was taken with a high sensitive camera (Olympus DP-72) operated on DP2BSW Software. 

## Oocyte preparation and *in vitro* maturation

The procedure of *in vitro* maturation (IVM) was performed as described previously (33). In brief, cumulus-oocyte complexes (COCs) were aspirated from antral follicles (2-8mm) of abattoir-derived ovaries using 18-gauge needlesattached to a vacuum pump (80 mmHg). COCs with homogeneous cytoplasm and more than three layers ofcumulus cells were then incubated for 24 hours in maturation medium [TCM-199 supplemented with 2.5 mM sodium pyruvate, 100 IU/mL penicillin, 100 µg/mL streptomycin, 1 mg/mL estradiol-17ß, 10 µg/mL follicle-stimulating hormone (FSH), 10 µg/mL luteinizing hormone (LH), 100ng/mL epidermal growth factor (EGF), 0.1 mM cysteamine, and 10% fetal calf serum (FCS)] at 38.5°C in a humidified atmosphere of 6% CO_2_ in air. 

## Intracytoplasmic sperm injection and artificial oocyte activation 

Labeld and decondensed sperm cells were used for ICSI according to Sekhavati et al. (9). Artificial activation of injected oocytes was performed according to Nasr- Esfahani et al. (34) with minor modifications. Briefly, 20 minutes after ICSI, oocytes were activated using calciumionophore (5 µM for 5 minutes) prepared in HEPES-tissue culture medium 199 (H-TCM99) plus 1 mg/ml BSA in the dark, followed by washing in H-TCM199 plus 3 mg/ ml BSA. Activated oocytes were incubated in a modified formulation of synthetic oviduct fluid (mSOF) left to rest for 3 hours before being incubated in 2 mM 6-dimethyl aminopurine (6-DMAP) for 4 hours. Oocytes cultured in mSOF medium at 38.5°C, 6% CO_2_, 5% O_2_, and maximum humidity for 8 days.

## *In vitro* RNA production and microinjection into the oocytes 

Capped phiC31 integrase RNA was generated by transcription of pUC19-phiC31polyA vector using the Transcript Aid^TM^ T7 High yield kit (Thermo, USA) and m7G(5´)ppp(5´)G RNA Cap (Biolabs, UK). The integrity of the RNA was assessed by electrophoresis on a 1% agarose gel. Before loading on the gel, the RNA was denatured by using the loading buffer provided in the Thermo kit according to the manufacturer’s instruction. PhiC31 RNA was diluted in microinjection TE buffer (10 mM Tris and 0.1 mM EDTA, pH=7.4) to a final concentration of 10 ng/µl. For microinjection, two rounds of oocyte microinjection were conducted. In first round, phiC31 RNA was injected into the cytoplasm of each MII oocyte. In the second round, completely decondensed sperm cells were incubated with pDB2 plasmidand used for microinjection into the oocyte. Microinjected oocytes were artificially activated and cultured for embryodevelopment as described above for ICSI oocytes. In this study, ICSI with non-transfected decondensed sperm cells was considered as control.

## Somatic cell preparation and transgenesis 

Primary bovine fetal fibroblast (BFF) cell line was established from a 65-day old female fetus conceived by natural mating as follows: primary bovine fetal fibroblast culture derived from a natural mating was established by isolating the cell from a 65-day old fetus. The skin of the fetus was extensively washed in Ca^2+^ and Mg^2+^ free phosphate buffer solution (PBS) containing 1% (v/v) of a cocktail of penicillin-streptomycin and amphotericin B. Then, the sample was cut into 2-3 mm pieces. The explants were cultured in Dulbecco’s modified Eagle medium F-12 (DMEM/F-12) containing 10% FCS, 1% penicillin- streptomycin and amphotericin B at 37°C in a humidified atmosphere of 5% CO_2_ until reaching 90-95% confluence. Cells were passaged twice, and then frozen, thawed and passaged in liquid nitrogen prior to transfection. 

The procedure of primary cell culture was as described previously (35). In brief, A population of 2×10^5^ BFF was cultured in 6-well tissue culture plates (Orange Scientific, Switzerland) containing DMEM/F-12 enriched with 10% FCS. BFFs at 60-70% confluence were co-transfected by 1 µg pDB2 and 1 µg EGFP-EF1 alpha plasmids with 3 µg pCMVInt (1:3 ratio) using Lipofectamine2000 (Invitrogen, USA) according to the manufacturer’s instruction. Six hours following transfection, the medium was changed with fresh culture medium for 24 hours before being distributed colony selection culture dishes (Falcon, 1005, Germany). Forty eight hours post culture, cells were treated with 400 µg/ml G418 for 21 days and developed colonies were isolated for subculture asdescribed previously. An established BFF line which was previously obtained by transfection of EGFP-OCT4 plasmid (www.royaninstitute.org) containing a neomycin resistance gene, via lipofectione2000 according to Jafarpuor et al. (35). To assess the expression of *EGFP* in colony cells derived following G418 treatment, cells were visualized and observed under a fluorescent microscope (Olympus BX51, Japan). To detect the nuclei, cell were stained with Hoechst 33342 before observation. The long term ectopic expression of *EGFP* in the first two cell lines was evaluated during 5 weeks following colony selection using fluorescence microscopy observation. These clones were trypsinized and subcultured to prepare a monolayer of stably transfected cells.

## Identification of pseudo attP sites and polymerase chain reaction screening for site specific integration in transfected cells 

To find possible new pseudo attP sites that could be recognized by phiC31 integrase, an inverse PCR (IPCR) approach was implemented as described. In brief, stably transfected bovine fibroblasts were harvested and the genomic DNA extracted by DNeasy Blood < Tissue Kit (Qiagen, Germany). Five µg of genomic DNA was digested with a couple of compatible enzymes, BglII and BamHI. The aforementioned enzymes recognize two different sites but produce similar cohesive ends. Both enzymes cut at least one site in PDB2. The digested fragments were extracted with phenol/chloroform and precipitated with ethanol. It was important to use low amounts of DNA for appropriate self-circulation of digested DNA in the ligation reaction for efficient inverse PCR. Thus, various amounts of DNA (0.5 to 5 ng) were prepared and used for ligation using DNA Ligation Kit (Takara, Japan) as describe in manufacturer protocol. The half-nested PCR was performed across the left junction of assumed recombination site. The circulated DNA was used as a template for the first round of PCR utilizing EGFP-F: 5´-ATGGTGAGCAAGGGCGAGGAG-3´ attB-F3: 5´-GTAGGTCACGGTCTCGAAGC-3´ primers. 1 µl of the first round PCR product was used in the second round of PCR utilizing attB-F3 and EGFP-F (nested): 5´-CGCACCATCTTCTTCAAGGACG-3´ primers. The PCR steps were conducted as follows: 94°C for 10 minutes as an initial denaturation step, followed by 35 repetitive cycles at 94°C for 30 seconds, 56°C for 4 minutes, and 72°C for 2 minutes. Final extension of 72°C for 5 minutes was performed at last stage of PCR and PCR products subjected to 1% (w/v) agarose. The obtained bands from IPCR were purified and ligated into T-vector and sequenced. To determine genomic location of pseudo attP, the obtained sequences were analyzed by BLAST search against bovine genome in various databases. PCR screening for detection of site specific recombination junction was carried out for 7 sites. Five sites were those previously reported (28-30) and two unknown sites which were detectedin the present study using IPCR. Nested primers were designed (Table 1). 

Approximately 10^3^ transfected cells were lysed by freezing/thawing and a nested PCR was performed for detection of site specific junction in transfected fibroblasts and colonies selected under antibiotic therapy. The first round of PCR (PCRI) program was as follows: 94°C for 5 minutes as initial step of denaturation, followed by repetitive 35 cycles of 94°C for 30 seconds; 55°C for 30 seconds; and 72°C for 20 seconds, and a final extension period of 72°C for 5 minutes. PCRI products were used as template for the second round of PCR (PCRII) with program as followed: 94°C for 5 minutes as initial step of denaturation, followed by repetitive 35 cycles of 94°C for 30 seconds; 62°C for 30 seconds; and 72°C for 20 seconds, and a final extension period of 72°C for 5 minutes. Direct sequencing was performed using automatic DNA sequencing method utilizing the same primers.

## Somatic cell nuclear transfer 

The process of zona-free SCNT was as described by Oback et al. (36) with minor modifications. In brief, denuded IVM oocytes were released from their zona pelucida by brief incubation (up to 45 seconds) in 5 mg/ ml pronase dissolved in HTCM199 containing 10% FCS. Enucleation of the oocytes were performed in phosphate buffer saline free of Ca^2+^ and Mg^2+^ (PBS) supplemented with 20% FCS, Na-pyruvate (2 mg/ml), BSA (1 mg/ ml), polyvinyl alcohol (PVA, 1 mg/ml) and glucose (0.036 mg/ml). Zona-free oocytes were incubated in enucleationmedium containing 5 µg/ml H33342 for 5 minutes before enucleation. Enucleation was carried out at ×100 magnification of a pre-warmed microscopic stage (Olympus, IX71, Japan) under UV exposure with the help of blunt perpendicular break enucleation pipettes (15-20 µm inner diameter). Nuclear transfer was carried out using three cell types: two cell lines in which *EGFP* gene was stably integrated into pseudo sites detected in this study and one EGFP-OCT4 cell line. For cell cycle synchronization at G0/G1, cells were cultured in presence of 0.5% FCS for 4-5 days. Immediately before nuclear transfer, a low density of somatic cells was prepared in a drop of HTCM199+0.5% FCS containing 10 µg/ml phytoheamoglutinin. Then, a group of 5 to 10 enucleated oocytes were added to the droplet and each oocyte was gently pushed attP over a single cell of population of cells synchronized in G0/G1 stage of cell cycle by serum starvation. The oocyte-donor cell couplets were placed between two electrodes (0.5 mm apart), overlaid with a hypo-osmotic fusion medium (0.2 M mannitol, 100 µM MgSO_4_, 50 µM CaCl_2_, 500 µM Hepes, 0.05% BSA) and aligned first manually and then by application of AC current (7 v/cm, 1000 kHz, for 10 seconds). Fusion was induced by two successive DC currents (1.75 kv/cm, 30 µseconds with 100 µseconds interval) fused couplets were kept in maturation medium containing 0.0 and 1.0 mM SAH for 1-2 hours. All fused embryos were further activated, in brief, embryos were incubated with 5 µM calcium-ionophore for 5 minutes followed by 4 hours exposure to 2 mM 6-dimethylaminopurine dissolved in TCM199 containing 10% FCS, 0.2 mg/ml PVA, 3 mg/ml BSA plus 0.0 and 1.0 mM SAH. Activated reconstituted oocytes were cultured in groups of ten in wells (36, 37) drained in 10 µl droplets of mSOF embryo culture medium at the same conditions described for ICSI embryos. On days three and seven after fusion, the reconstructed embryos were checked for cleavage and blastocyst rates, respectively. In this study, SCNT with non-transfected somatic cells was considered as control. 

## Polymerase chain reaction screening for site specific integration in transgenic embryos 

For screening of site specific recombination junctions in transgenic embryos, *EGFP* positive embryos (selected using fluorescent microscope) at day 8 of embryo development were pooled and lysed by freezing/thawing. Nested PCR was performed for detection of seven site specific recombination junction. 

## RNA extraction and reverse transcription

Total mRNA was extracted from blastocysts at day 8 of embryo culture using the RNeasy Micro Kit (Qiagen^TM^, Germany) and subsequently, cDNAwas synthesized by the RevertAid^TM^ First Strand cDNA Synthesis Kit (Thermo, USA) according to their manufacturer’s recommendation.

## Real-time polymerase chain reaction 

To detect the presence of *EGFP* mRNA in SMGT derived embryos, real-time polymerase chain reaction (RT-PCR) was conducted using 

*rEGFP*-F: 5´-CAAGCAGAAGAACGGCATCAAG-3´ *rEGFP*-R: 5´GTGCTCAGGTAGTGGTTGTC-3´ primers. In this regards, cDNA from SMGT derived embryos were subjected to RT-PCR with following programs: 94°C for 5 minutes as an initial denaturation step, followed by 35 repetitive cycles at 94°C for 30 seconds, 60°C for 30 seconds, and 72°C for 20 seconds. Final extension of 72°C for 5 minutes was performed at last stage of PCR.

## Results

### Identification of phiC31-mediated recombinant sites in bovine genome

**Fig.2:**
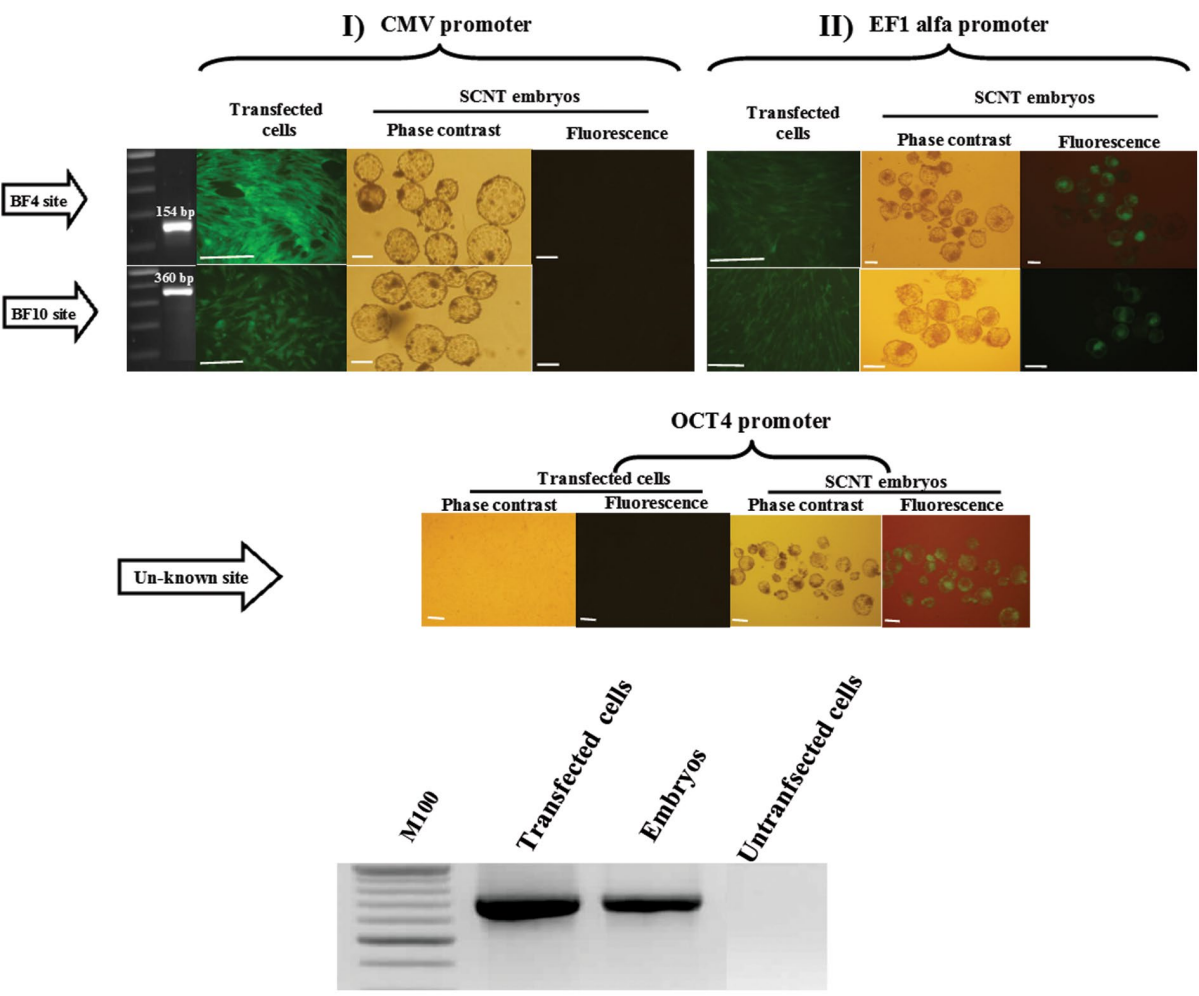
Microscopic observation in bovine fibroblast cells and by somatic cell nuclear transfer (SCNT) derived embryos. A. *EGFP* expression under two different promoters regulation in transgenic bovine fibroblast cells and SCNT derived embryos which obtained by phiC31 integrase systems in BF4 and BF10 pseudo sites, I. From left to right, column 1; Nested PCR product for detection of the BF4 and BF10 sites, column 2; Targeted stable transgenic bovine in BF4 and BF10 sites which obtained by co-transfection of pCMVInt and pDB2, column 3 and 4; Phase contrast and fluorescence microscopic observation from SCNT embryos obtained by BF4 and BF10 targeted bovine fibroblast cells, respectively, II. Nested PCR product for detection of the BF4 and BF10 sites, column 2; Targeted stable transgenic bovine in BF4 and BF10 sites which obtained by co-transfection of pCMVInt and EGFP-Ef1 alpha, column 3 and 4; Phase contrast and fluorescence microscopic observation from SCNT embryos obtained by BF4 and BF10 targeted bovine fibroblast cells, respectively, B. *EGFP* expression under OCT4 promoters in transgenic bovine fibroblast cells and its SCNT derived embryos according to Jafarpour et al. (35) column 1 and 2; Phase contrast and fluorescence microscopic observation of EGFP-OCT4 cell line, respectively and column 3 and 4; Phase contrast and fluorescence microscopic observation from SCNT embryos obtained by EGFP-OCT4 cell line, and C. Genomic PCR for amplification of complete *EGFP* Open Reading Frame in *EGFP* positive bovine cells and embryos (scale bars: 100 µm).

**Fig.3:**
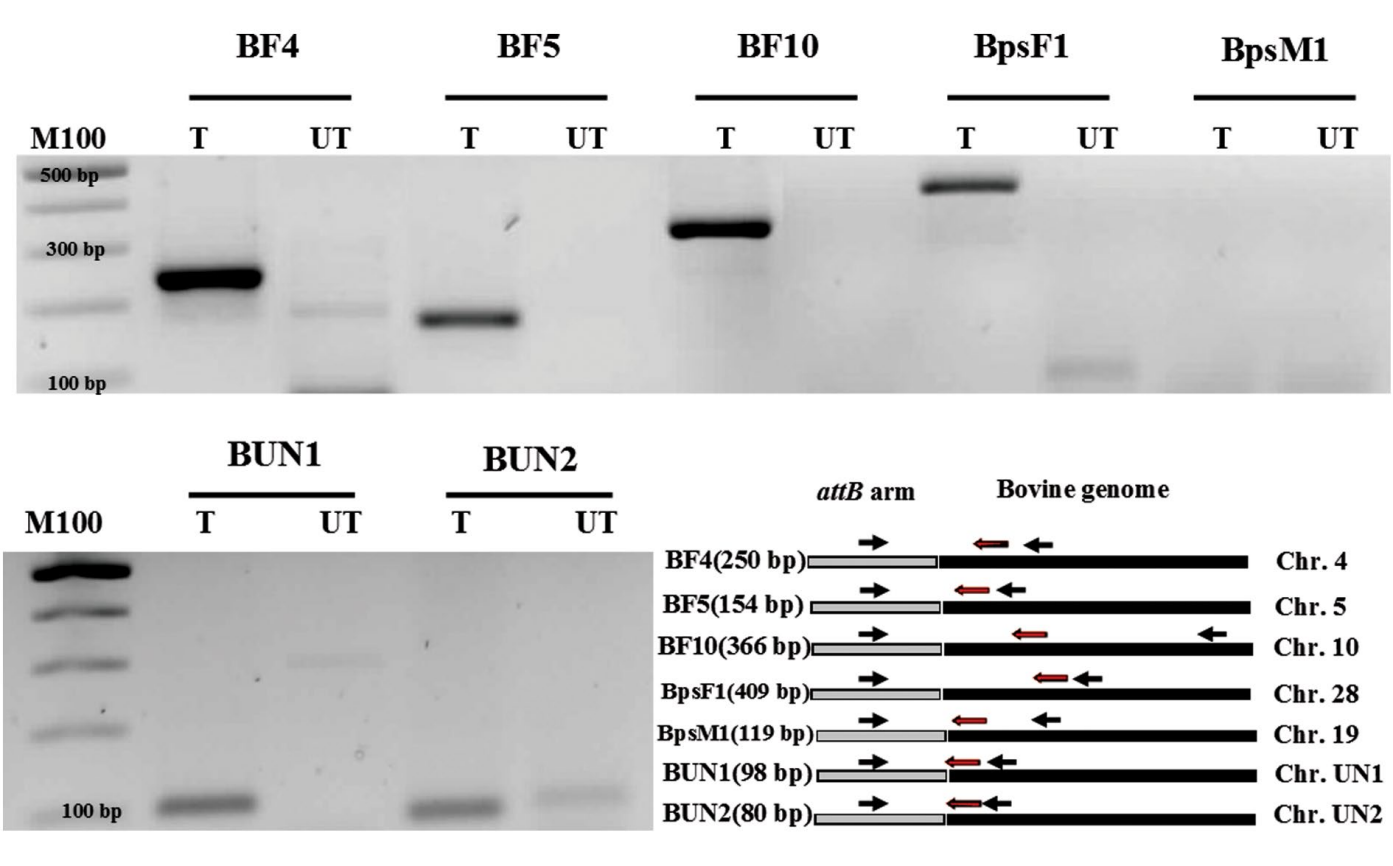
Identification of recombinant sites created by phiC31 integrase in bovine genome. T; Transfected bovine fibroblast and UT; Untransfected bovine fibroblast.

Bovine fibroblasts after successful co-transfection with pCMVInt and pDB2, EGFP-EF1 alpha showed constant *EGFP* expression throughout 11 passages (Fig .2). IPCR detected three new pseudo attP sitesin bovine genome which were named BF5, BUN1 and BUN2 with 32, 16 and 48% of identity with wild type of attP sequence. To screen the recombinant sites which were mediated by phiC31 integrase, we designed nested primers for BF5, BUN1 and BUN2 as well as four other recombinant sites which previously reported in bovine genome (Table 1) (28, 29). Nested PCR amplified whole expected recombinant sites with exception of BpsM1 site in co-transfected bovine fibroblasts by pCMVInt and pDB2 vectors (Fig .3). During colony selection procedure, only two individual cell clones with *EGFP* integrated into the BF4 and BF10 sites were selected for each co-transfection strategy (CMVInt-pDB2 and CMVInt-*EGFP* EF1 alpha) (Fig .2). 

### Vector assay integration 

To evaluate the phiC31 integrase functionality, we used pBCPB^+^ as an intra-molecular assay vector. This vector carries phiC31 attP and attB sites in direct orientation flanking with a LacZ gene. SincephiC31 integrase has a precise target recombinant activity; it can distinguish two att sites on pBCPB^+^ and delete the LacZ sequence. The recombinant site could be identified by a PCR reaction with specific primers that can amplify a 401 bp product as a detector for phiC31 site specific activity. The positive recombination control was performed by *in vitro* incubating pBCPB^+^ vector with a crude protein extract from integrase-expressing *E.coli* (20). *In vitro* produced phiC31 protein had precise activity and PCR reaction amplified an expected 401 bp product when reaction buffer was used as a template. In parallel, PCR on co-injected oocytes by phiC31 mRNA and pBCPB^+^ also amplified an expected 401 bp product, indicating that recombination has occurred for integrase-mediated site- specific recombination between attP and attB. Therefore, *in vitro* produced phiC31mRNA had accurate activity in bovine oocyte cytoplasm. 

### Exogenous DNA uptake by heparin-glutathione pretreatedsperm 

After incubation with exogenous DNA, sperm cells were treated with heparin-GSH which resulted in three types of decondensation pattern according to Sekhavati et al. (9). Assessment of these decondensed sperm cells under fluorescent microscope indicated that spontaneous uptake of labeled pDB2 plasmid was mostly confined to the head of spermatozoa. The pattern of DNA uptake was observed either in acrosomal ridge or in the post-acrosomal region (Figes.1A, 3). Interestingly, DNA uptake was not influenced by the degree of sperm head decondensation (data not shown).

### Sperm mediated gene transfer and enhanced green fluorescent protein expression assessment 

*EGFP* expression was not detected after fluorescent assessment of SMGT embryos obtained by injection of sperm cells exposed to 200 or with 1000 ng of exogenous pDB2 DNA. Microscopic observations showed that morphological aspects of the SMGT-derived embryos were not influenced by exogenous DNA concentration (Fig .1B). However, *EGFP* expression was detected at mRNA level by real-time polymerase chain reaction (RTPCR) in SMGT-derived embryos of both groups (Fig .1C). 

### Targeted sperm-mediated gene transfer 

**Fig.4:**
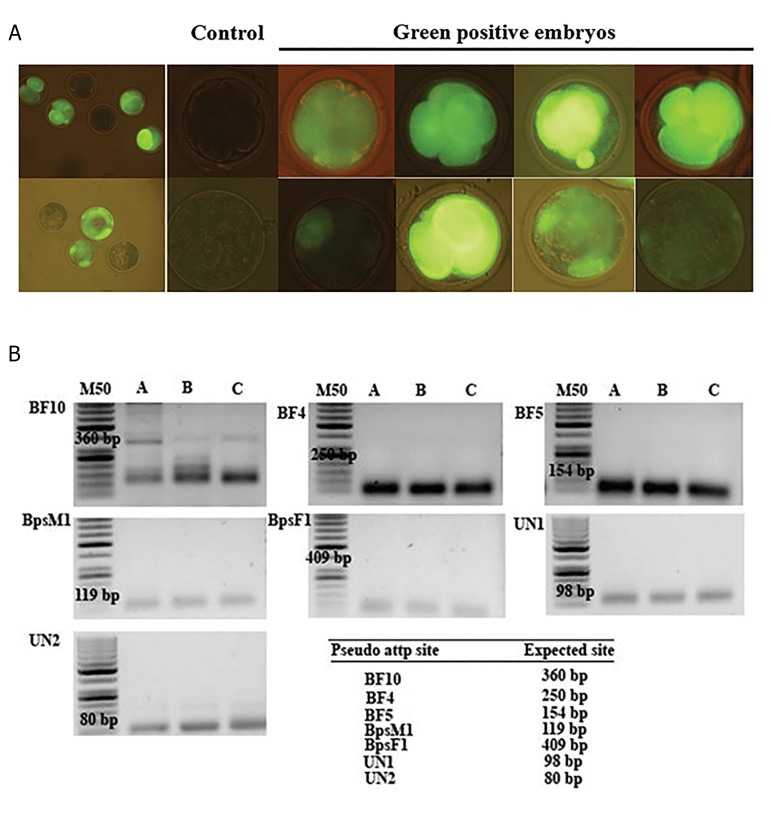
Targeted sperm mediated gene transfer using phiC31 system and PCR screening for detection of recombinant junction. A. Green positive bovine TSMGT derived embryos in deferent stage of pre-implantation development and B. Nested PCR screening for identifying the likely recombinant junction which could be created by phiC31 integrase system. A; Green positive bovine TSMGT derived embryos, B; SMGT derived embryos, and C; Negative control without DNA in PCR reaction.

PhiC31 mRNA may be transcribed in the cytoplasm using cytoplasm transcription machinery and subsequently active integrase could import into the nucleus where it would possibly catalyze the target gene in the bovine pseudo attP sites. Because the recombinant sites attR and attL are not substrate for the phiC31 integrase, the reaction is unidirectional. TSMGT combined with microinjection of phiC31 mRNAresulted in eight *EGFP* positive embryos out of 310 oocytes injected (approximately: 2.5%). The majority of TSMGT-derived embryos had low quality but the intensity of their fluorescent *EGFP* was considerable (Fig .4A). The results of PCR screening for seven possible recombinant junctions, which could be generated by phiC31 integrase system in *EGFP* positive embryos, showed that just BF10 pseudo attP site was amplified in the expected size (Fig .4B). Subsequently, sequencing the amplified fragment confirmed the presence of BF10 pseudo site in pooled *EGFP* positive derived embryos by TSMGT.

### Targeted somatic cell nuclear transfer-mediated gene transfer 

*EGFP* signal was clearly observed in fibroblasts transfected with either CMV-EGFP or EF1-EGFP vectors (Fig .2).Targeted transfection had no apparent effect on the competence of the reconstituted oocytes to cleave and to further develop to the blastocyst stage compared to control (Table 2). *EGFP* signal i. Was not detected in any stage of SCNT embryos reconstructed with CMV-EGFP transgenic fibroblasts, ii. Was clearly observed throughout *in vitro* development of EF1EGFP reconstructs, and iii. Was observed only after 8-16 cell stage in SCNT embryos reconstructed with OCT4-EGFP transgenic fibroblasts. 

**Table 2:** The rate of cleavage and blastocyst formation in SCNT derived embryos using transfected and non-transfected cells


Treatment	n	Cleavage (%)	Blastocyst (%)

SCNT control (non-transfected)	108	91 (84.26 ± 4.5)	30 (32.97 ± 5.3)
SCNT transfected (BF10 )	135	122 (90.4 ± 6.1)	34 (27.87 ± 3.9)
SCNT transfected (BF5)	125	107 (85.5 ± 4.7)	32 (29.99 ± 3.9)


SCNT; Somatic cell nuclear transfer and BF10; Bovine fibroblast chromosome10.

## Discussion

This study introduced that the two new bovine pseudo attP sites are in favor of site directed transgene expression. It was also demonstrated that phiC31 integrase can be used for production of transgenic bovine embryos by SCNT and SMGT techniques. Connected to two most routine techniques of animal transgenesis site-specific transgenesis system for efficient generation of bovine embryos carrying targeted reporter gene. Therefore, these results in agreement with other studies in bovine (28-31) show that attP inclusion into a selection cassette can be used as a powerful tool to conduct site directed transgenesis in mammalian cells and embryos. 

In our study, completely decondensed sperm still had the ability to store the exogenous DNA. By incubation of sperm cells decondensed with an egg extract with linear DNA, Ishibashi et al. (38) successfully produced transgenic Xenopus. Even though, our results revealed that sperm chromatin decondensation with heparin-GSH improved development of ICSI-SMGT embryos (data not shown), *EGFP* expression was detected only at mRNA level but there was no any detectable EFGP protein in blastocyte. This is consistent with the previous reports that SMGT- derived bovine embryos are not able to express *EGFP* protein and only *EGFP* mRNA is detectable (38, 39).

When cytoplasmic injection of phiC31 mRNA was carried out with the aim of gene targeting before TSMGT,only 8 green positive embryos out of 310 oocytes injected. This low efficiency of is compatible with the report of Hoelker et al. (40) who detected only 3.6% transgenic bovine embryos following conventional SMGT. Screening for recombinant junction by nested PCR amplified BF10 junction in TSMGT-derived embryos. This pseudo attP site was previously reported by Qu et al. (29) as a preferred site recognized by phiC31 integrase in bovine genome. Sequencing also confirmed the presence of BF10 junction sequence in TSMGT derived embryos. It has been shown that the half-life of phiC31 integrase in liver cells is about 6 hours and a small fraction of active integrase may gain access to the nucleus, whereas the bulk being cytoplasmic (40, 41). So, it is likely that the injected phiC31 mRNA could be translated into protein and reached to the nucleus for site specific recombination of donor plasmid harboring attB into bovine pseudo attP sites. Indeed, it seems that phiC31 integrase system has a proper potential for gene targeting in the SMGT protocol.

To investigate whether the *EGFP* expression is resulted from stable gene integration or extra chromosomal expression, we carried out SCNT experiments with stable targeted (for BF10 and BF5 sites) transgenic (for CMVEGP and EF1-EGFP) fibroblasts. Interestingly, none of the cloned embryos showed *EGFP* expression under CMV promoter regulation but *EGFP* was expressed successfully and efficiently under EF1 promoter regulation. Control SCNT embryos carrying EGFP-OCT4 showed *EGFP* signals only at the morula and blastocyst stages. This results may lend support for the notion that de novo methylation of pre-implantation embryo development can silence integrated viral DNA (32, 41). It seems that green- positive embryos derived by TSMGT have expressed *EGFP* as an extra-chromosomal gene which remained unaccessible to the de novo methylation machinery of embryo while remains non-integrated (32). Accordingly, detection of *EGFP* signal in almost all cloned embryos carrying EF1-EGFP and OCT4-EGFP could be due to the lack of any viral DNA in their plasmid structure.

## Conclusion

PhiC31 has been successfully used for site-directed transgenesis in a variety of tissues and organs under in vivo and ex vivo conditions in several species. This study introduced that the two new bovine pseudo attP sites are in favor of site directed transgene expression. It was also demonstrated that phiC31 integrase can be used for production of transgenic bovine embryos by SCNT and SMGT techniques. Therefore, these results in agreement with other studies in bovine show that attP inclusion into a selection cassette can be used as a powerful tool to conduct site directed transgenesis in mammalian cells and embryos.
